# Emerging causes of anticancer therapies−induced Stevens-Johnson syndrome and toxic epidermal necrolysis: evidence from disproportionality analysis of the FDA adverse event reporting system

**DOI:** 10.3389/fimmu.2025.1646038

**Published:** 2025-08-27

**Authors:** Wensheng Liu, Xue Song, Qiong Du, Jiyong Liu

**Affiliations:** ^1^ Department of Pharmacy, Fudan University Shanghai Cancer Center, Shanghai, China; ^2^ Department of Oncology, Shanghai Medical College, Fudan University, Shanghai, China; ^3^ Clinical Research Center, Renji Hospital, Shanghai Jiao Tong University School of Medicine, Shanghai, China

**Keywords:** cancer, Stevens-Johnson syndrome, toxic epidermal necrolysis, pharmacovigilance, disproportionality analysis, FAERS

## Abstract

**Background:**

Stevens-Johnson syndrome (SJS) and toxic epidermal necrolysis (TEN) are potentially fatal cutaneous adverse events of drug treatment. Evidence for SJS/TEN risk from current anticancer therapies in population-based studies is scarce.

**Objective:**

The present study aims to characterize the profiles and risk factors of SJS/TEN related to contemporary anticancer regimens.

**Methods:**

Reported odds ratios (ROR) were employed to identify anticancer drugs associated with SJS/TEN development using FAERS data from January 2004 to September 2024. Single factor, LASSO, and multivariable logistic regression analysis were performed to explore the risk factors of SJS/TEN related to anticancer therapies. Weibull shape parameter analysis was applied to the onset time of reported SJS/TEN.

**Results:**

A total of 3471 unique SJS/TEN events were identified for 159 anticancer drug pairs, of which 31 drugs were identified as significantly disproportionate. Targeted therapies accounted for 35.93% of pairs, chemotherapies for 35.52%, and immunotherapies for 21.52%. The median onset time of SJS/TEN with anticancer therapies was 17 days. Moreover, multivariable logistic regression showed that age exceeding 65, female gender, and 10 anticancer drugs were significant risk factors for anticancer therapy-related SJS/TEN.

**Conclusions:**

This study provides real-world evidence regarding the burden of SJS/TEN associated with anticancer therapies. Addressing this knowledge gap will facilitate the optimization of clinical management for SJS/TEN. Further research to establish causality and inform clinical decision-making related to anticancer therapy-associated SJS/TEN is urgently needed.

## 1 Introduction

Stevens-Johnson syndrome (SJS) and toxic epidermal necrolysis (TEN) are life-threatening dermatologic emergencies characterized by widespread keratinocyte apoptosis leading to epidermal detachment and mucosal erosions (1, 2). Global epidemiological data indicate an annual incidence of 2–7 cases per million population for SJS/TEN (3). Though diagnostic awareness has improved and supportive care protocols have been standardized, mortality persists at 15-30% in severe cases, primarily attributed to sepsis-induced multiorgan failure in the acute phase (4, 5). Survivors frequently experience chronic sequelae, including vision-threatening ocular surface disease and pulmonary dysfunction, substantially impacting long-term quality of life (6). Previous studies confirm that over 85% of SJS/TEN cases involve exposure to high-risk medications (particularly antiepileptics, antibiotics, and nonsteroidal anti-inflammatory drugs), mandating immediate drug discontinuation alongside multidisciplinary critical care as the cornerstone of management (4, 7–9).

In the context of cancer treatment, traditional chemotherapy agents, like capecitabine, carboplatin, and paclitaxel, have long been recognized as potential culprits for SJS/TEN (10, 11). Recently, the emergence of novel anticancer agents, particularly immunotherapies and targeted therapies, has revolutionized the traditional cancer treatment paradigm, but the burden, risk and characterization of SJS/TEN associated with these treatments have also become an increasingly important area of concern (12–15). Additionally, the revolutionary chimeric antigen receptor T-cell therapy, which modifies a patient’s own T cells to target cancer cells, has also raised concerns regarding the potential risk of SJS/TEN (16). Otherwise, cancer patients often have comorbidities, multi-system involvement, and receive multiple medications and treatments, all of which contribute to the heightened intricacies and challenges encountered in the management of SJS/TEN within the context of anticancer therapies. Hence, conducting large-scale population-level pharmacoepidemiological investigations to delineate the causal relationship between specific anticancer agents and the risk of SJS/TEN is critical for balancing tumor efficacy and toxicity remission.

Pharmacovigilance databases can serve as an essential complementary source to investigate SJS/TEN prevalence and the structure of suspected anticancer drugs (12). FDA Adverse Event Reporting System (FAERS) is a global, authoritative, open-source database for the spontaneous reporting of adverse events (AEs) that collected in the post-marketing routine clinical care (7–9). By leveraging the large-scale data in FAERS, the present study aims to quantify SJS/TEN risks across anticancer drug classes and identify high-risk subpopulations, thereby providing a vigilant reference for clinical decision-making.

## 2 Materials and methods

### 2.1 Data source and pre-processing

The FAERS, which utilizes the Medical Dictionary for Drug Regulatory Activities (MedDRA) preferred terms (PTs) for standardized coding of AEs, operates as a vital surveillance platform for reporting and analyzing adverse drug reaction data. Data sources for this study are freely available from the FDA website (https://fis.fda.gov/extensions/FPD-QDE-FAERS/FPD-QDE-FAERS.html). These reports encompass patient demographics (DEMO), medication use (DRUG), therapy start and end dates for the reported drugs (THER), indications (INDI), AEs (REAC), outcomes (OUTC), and report sources (RPSR). Duplicate reports were excluded using FDA-recommended methods: (i) for reports with multiple PRIMARYIDs, only the record with the latest FDA_DT was retained; (ii) for those with identical CASEID and FDA_DT, the one with the largest Primary value was kept; (iii) for reports lacking unique or reliable identifiers, duplicates were identified via key fields, including patient information, drug information, adverse event information, and report date; (iv) uncertain cases underwent manual review to ensure accurate deduplication.

This retrospective real-world pharmacovigilance study utilized reports retrieved from the FAERS database spanning from 1 January 2004 to 31 September 2024. Since the FAERS databases are publicly accessible, anonymized, and de-identified, neither informed consent nor ethical approval was necessary. To guarantee methodological transparency and reproducibility, this study strictly adhered to the READUS-PV guideline (17, 18).

### 2.2 Identification of SJS/TEN reports associated with anticancer drugs in FAERS

In this study, narrow-scope PTs in the Standardized MedDRA Query (SMQ) were employed to identify SJS/TEN reports in FAERS, which were investigated using the MedDRA (Version 25.0) terms: Stevens-Johnson syndrome (10042033), toxic epidermal necrolysis (10044223), and SJS-TEN overlap (10083164). To enhance data accuracy and reliability, the drug names recorded in the DRUG file were standardized through the utilization of the Drugs@FDA platform (https://www.accessdata.fda.gov/scripts/cder/daf/index.cfm), which facilitated the conversion of brand names into generic names. Additionally, a case was included only when the drug was designated as a “primary suspect” for SJS/TEN.

### 2.3 Risk factors for SJS/TEN associated with anticancer drugs

The independent variables evaluated included gender (with females as the reference group), age (with the <65 years group as the reference), and drug (with all other anticancer drugs as the reference group). For patient demographic characteristics (including gender and age), only reports containing complete data fields were included in the final cohort. Data quality control measures involved the exclusion of physiologically implausible values, specifically age records exceeding 120 years and weight measurements surpassing 400 kg.

Framework of regression analysis comprised three sequential phases: (1) Univariate Screening: The selection criteria required: the lower limit of the 95% CI of the reporting odds ratio (ROR025) > 1; Case count ≥ 50; adjusted *p*-value < 0.01. (2) Feature Selection: Suspect anticancer drugs demonstrating significant associations (*p* < 0.01) in the initial screening underwent Least Absolute Shrinkage and Selection Operator (LASSO) regression with 10-fold cross-validation to mitigate multicollinearity and optimize variable selection. (3) Multivariate Modeling: A logistic regression model incorporating LASSO-selected medications and baseline demographic variables was subsequently constructed to delineate independent risk factors for anticancer drug-associated SJS/TEN. The impact of each risk factor was quantified using odds ratios (ORs) and their corresponding 95% CIs, while statistical significance was assessed through parameter estimates, standard errors (Std. Error), z-values, and p-values.

### 2.4 Time-to-onset analysis

Time-to-onset duration from the FAER database was calculated from the time of the patient’s first prescription to the occurrence of the AEs. (AE onset time = EVENT_DT - START_DT, where EVENT_DT represents the date of AE occurrence and START_DT denotes the drug start date. Records with errors, specifically those in which EVENT_DT preceded START_DT, were excluded from the analysis). The median duration, quartiles, and Weibull shape parameter test were used to evaluate the time-to-onset data (19). In this study, the shape parameter β of the Weibull distribution was used to describe the varying incidence of SJS/TEN (i.e., changes in risk over time) and evaluate hazard functions for detecting SJS/TEN.

The Weibull shape parameter β determines three distinct failure patterns based on its 95% CI (**Supplementary Figure 1** shows the relationship between the Weibull distribution parameter -β values and failure patterns) (19, 20):

1. When the upper limit of CI is below 1 (β < 1), it suggests an early failure pattern with a hazard rate that initially increases then decreases;
2. When the CI encompasses 1, it indicates a constant hazard rate characteristic of random failures;
3. When the lower limit of CI exceeds 1 (β > 1), it demonstrates a wear-out failure mode with a monotonically increasing hazard rate over time.

### 2.5 Statistical analysis

Disproportionality analysis, employing the ROR, was conducted to evaluate the potential risk of SJS/TEN linked to novel anticancer drugs, with all other drugs/events documented in the FAERS database serving as the comparator. As shown in **Supplementary Table S1**, the RORs for the anticancer drugs related to SJS/TEN were calculated by using a two-by-two contingency table. The calculation formula of the ROR value and its 95% confidence interval (CI) was as follows:


$$\text{ROR}=\frac{\left(a/c\right)}{\left(b/d\right)}=\frac{ad}{bc}$$

$$\text{ROR}025={\text{e}}^{\text{ln}\left(\text{ROR}\right)-1.96\sqrt{\;\left(\frac{1}{\text{a}}+\frac{1}{\text{b}}+\frac{1}{\text{c}}+\frac{1}{\text{d}}\right)\;}}$$

$$\text{ROR}075={\text{e}}^{\text{ln}\left(\text{ROR}\right)+1.96\sqrt{\;\left(\frac{1}{\text{a}}+\frac{1}{\text{b}}+\frac{1}{\text{c}}+\frac{1}{\text{d}}\right)\;}}$$

For ROR, the ROR025 > 1 with at least three cases indicates a significant signal, which means the drug of interest may have the potential risk to induce the AE of interest.

An applied descriptive analysis was made based on the clinical characteristics of patients with SJS/TEN associated with anticancer therapies in the FAERS database. Independent risk factors for anticancer drug-related SJS/TEN were screened using multivariate regression and Bonferroni correction. All data management, statistical analysis, and graphical representations in this study were executed using R software, version 4.3.3.

## 3 Results

### 3.1 Clinical characteristics and burden of patients with SJS/TEN related to anticancer therapy in the FAERS dataset

**Figure 1:**
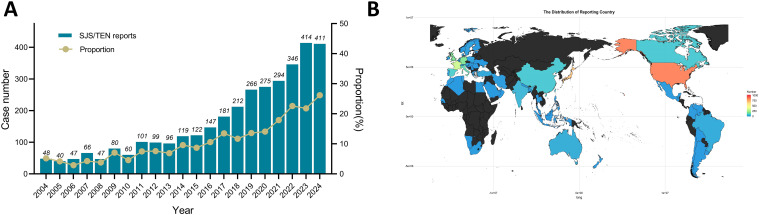
Statistics of SJS/TEN reports related to anticancer therapies in the FAERS from 2004 to 2024Q3. **(A)** Bar chart showing the number of SJS/TEN reports related to anticancer therapies per year, and a line chart showing the proportion of SJS/TEN reports associated with anticancer therapies as a percentage of all treatment-related SJS/TEN reports per year. **(B)** A heat map chart showing the number of SJS/TEN reports related to anticancer therapies between different reporting countries.

Detailed baseline characteristics of the included reports are summarized in **Table 1**. A total of 3471 reports were reported to have experienced SJS/TEN related to anticancer therapy in the FAERS database from January 2004 to September 2024. The median age of the subjects was 64 years (interquartile range [IQR] 52-71), with females comprising the majority at 46.1%. The median weight was 71 kg (IQR: 57-164). The most common underlying tumors were non-small cell lung cancer [n = 184, (5.3%)], breast cancer [n = 145, (4.2%)], and metastatic malignant melanoma [n = 93, (2.7%)]. In terms of report submission, health care professionals (84.8%) were the leading reporters. The primary outcomes for these subjects were concentrated in “death” (32.0%) and “hospitalization” (30.9%). The incidence of SJS/TEN associated with anticancer therapies has demonstrated progressive annual increases during the current decade (**Figure 1A**), and the United States [n = 858, (24.72%)], Japan [n = 705, (20.31%)], and France [n = 327, (9.42%)] were the leading reporting countries (**Figure 1B**).

**Table 1:** Demographic characteristic of anticancer drugs related Stevens-Johnson syndrome and toxic epidermal necrolysis for patients reported in the FAERS Database.

Characteristics		SJS/TEN reports (N=3471)
Age, years		
	< 18	172 (5.0%)
	18-65	1301 (37.5%)
	> 65	1151 (33.2%)
	Unknown	847 (24.4%)
	Median [Q1, Q3]	64 [52,71]
Gender		
	Female	1600 (46.1%)
	Male	1472 (42.4%)
	Unknown	399 (11.5%)
Weight, kg		
	< 50	131 (3.8%)
	50-100	710 (20.5%)
	> 100	333 (9.6%)
	Unknown	2297 (66.2%)
	Median [Q1, Q3)]	71 [57, 164]
Indication (Top Five)		
	Non-small cell lung cancer	184 (5.3%)
	Breast cancer	145 (4.2%)
	Metastatic malignant melanoma	93 (2.7%)
	Malignant melanoma	79 (2.3%)
	Renal cell carcinoma	75 (2.2%)
Reporter		
	Health-care professional	2944 (84.8%)
	Non-health-care professional	411 (11.8%)
Outcome		
	Death	1111 (32.0%)
	Disability	18 (0.5%)
	Life-threatening	447 (12.9%)
	Hospitalization	1072 (30.9%)
	Required intervention to prevent permanent impairment	4 (0.1%)
	Other Serious	802 (23.1%)

SJS/TEN, Stevens-Johnson syndrome and toxic epidermal necrolysis; Unknown, Missing data.

### 3.2 The percentage distribution of anticancer therapy involved in SJS/TEN development

**Figure 2:**
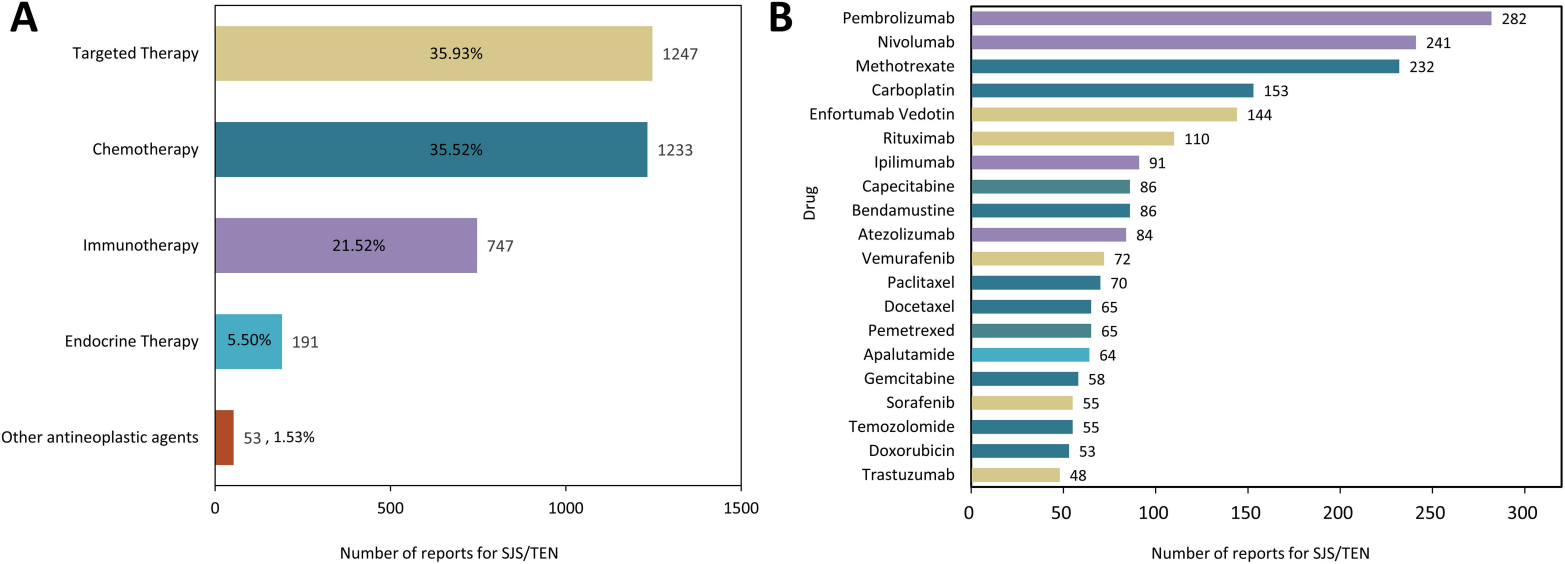
The distribution of anticancer therapy involved in SJS/TEN development. **(A)** Proportional distribution of SJS/TEN reports across different anticancer therapies. **(B)** Top 20 anticancer drug related to SJS/TEN development.

As listed in **Supplementary Table 2**, a total of 159 anticancer drugs have been reported to be associated with SJS/TEN development in the course of their application. Among them, targeted therapy exhibited the highest incidence (n = 1247, 35.93%), followed by traditional chemotherapy (n = 1233, 35.52%) and immunotherapy (n = 747, 21.52%) (**Figure 2A**). Of the top 20 anticancer therapeutics associated with the development of SJS/TEN, immune checkpoint inhibitors (ICIs) were the most frequently reported medications, comprising pembrolizumab (n = 282, 8.12%), nivolumab (n = 241, 6.94%), ipilimumab (n = 91, 2.62%), and atezolizumab (n = 84, 2.42%). This was followed by antimetabolites, including methotrexate (n = 232, 6.68%), capecitabine (n = 82, 2.48%), pemetrexed (n = 65, 1.87%), and gemcitabine (n = 58, 1.67%). Next were platinum compounds, carboplatin (n = 153, 4.41%) and antibody-drug conjugate, enfortumab vedotin (n = 144, 4.15%) (**Figure 2B**).

### 3.3 Disproportionality analysis: SJS/TEN induced by anticancer therapy

**Figure 3:**
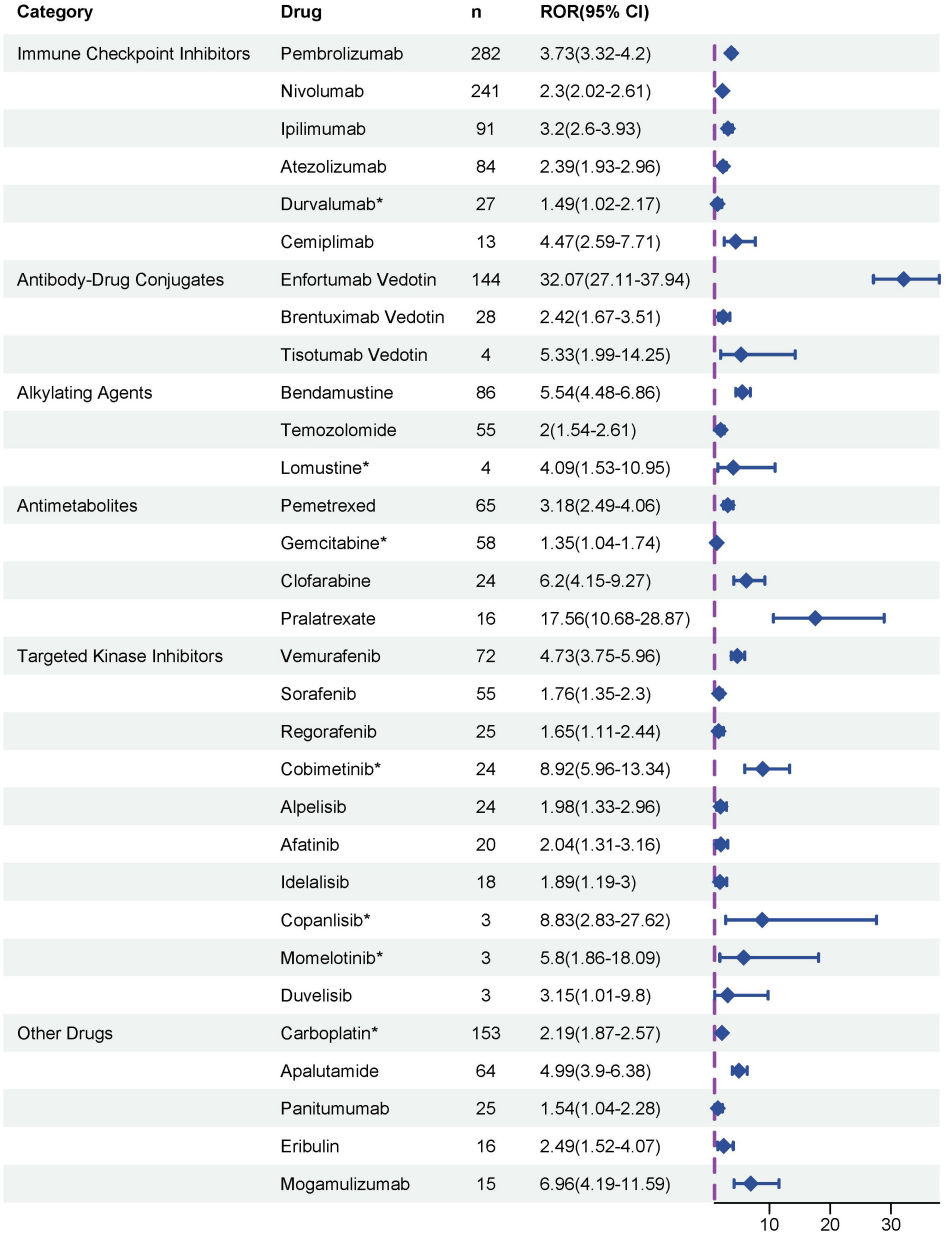
Disproportionality analysis of significant SJS/TEN reports associated with anticancer therapies. * indicated the alerts of SJS/TEN do not include in the drug package inserts.

To evaluate potential associations between anticancer therapies and the development of SJS/TEN, disproportionality analyses were performed on all reported drugs, and ROR was used to quantify the strength of the association. Ultimately, as depicted in **Figure 3**, a total of 31 anticancer drugs were identified as significantly associated with SJS/TEN development. In terms of signal strength, enfortumab vedotin had the highest ROR value, reaching 32.07 (95% CI 27.11 - 37.94), indicating a significant association with SJS/TEN development. Other top five anticancer drugs with strong positive signals were pralatrexate (ROR = 17.56, 95% CI 10.68 - 28.87), cobimetinib (ROR = 8.92, 95% CI 5.96 - 13.34), copanlisib (ROR = 8.83, 95% CI 2.83 - 27.62), mogamulizumab (ROR = 6.96, 95% CI 4.19 - 11.59), and clofarabine (ROR = 6.2, 95% CI 4.15 - 9.27), which warrant clinical attention. Furthermore, among these 31 anticancer drugs, the package inserts of 24 drugs contain warnings regarding SJS/TEN risks, while the remaining 7 drugs’ package inserts do not include SJS/TEN risk alerts. These seven drugs without SJS/TEN warnings are: carboplatin, gemcitabine, durvalumab, cobimetinib, lomustine, momelotinib, and copanlisib.

### 3.4 Risk factors for anticancer therapy-related SJS/TEN

**Figure 4:**
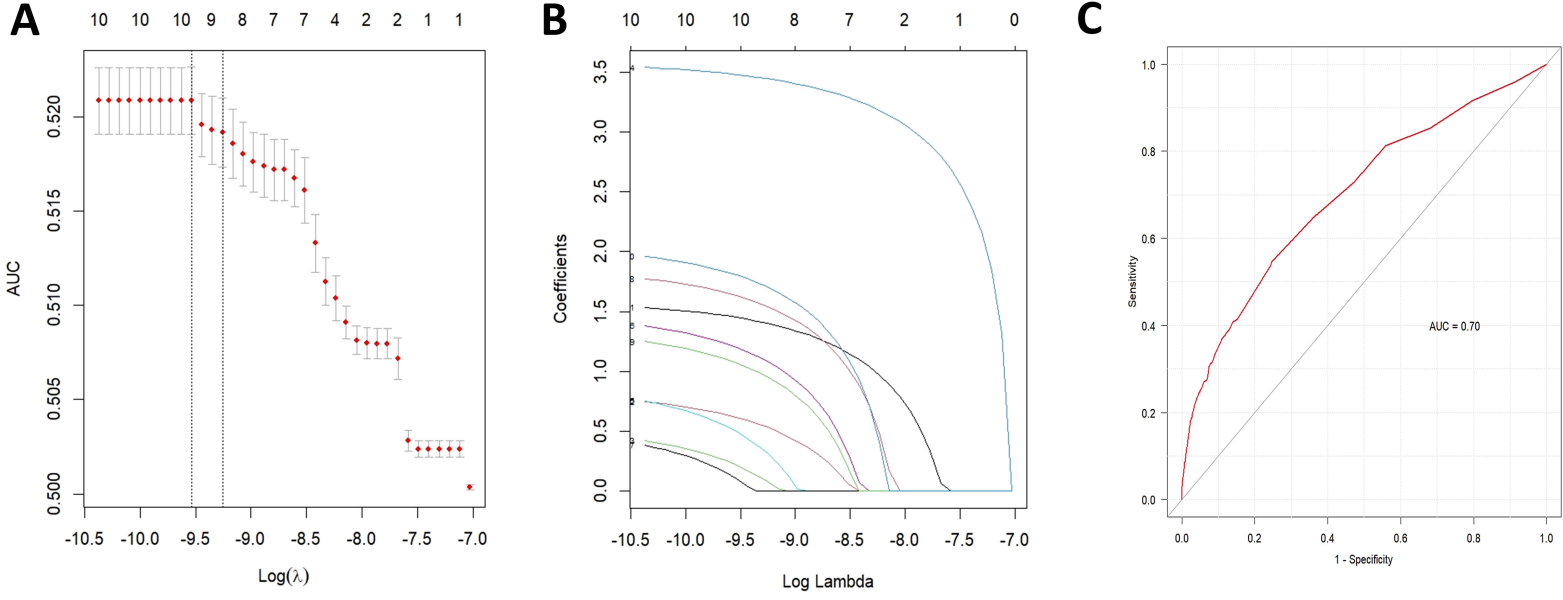
Results of the LASSO regression analysis and ROC curves for risk factors associated with SJS/TEN related to anticancer therapies. **(A)** Optimal regularization parameter λ in the LASSO regression model; **(B)** Evolutionary trajectories of variable coefficients across the λ-spectrum; **(C)** AUC of the multifactorial logistic regression analysis. LASSO, least absolute shrinkage and selection operator; ROC, receiver operating characteristic; AUC, area under curve; SJS/TEN, Stevens-Johnson syndrome and/or toxic epidermal necrolysis.

Potential drug candidates were initially selected based on the number of case reports > 50, a ROR025 exceeding 1, and a *p*-adjusted < 0.01. These candidates subsequently underwent univariate analysis. Drugs demonstrating significant associations (*p* < 0.01) in the initial screening were further analyzed using LASSO regression, resulting in the identification of 10 anticancer agents (**Figures 4A, B**). A multivariate logistic regression model was then constructed incorporating both these medications and relevant patient demographics (**Table 2**). Key results showed that patients aged over 65 years and female gender were significantly more likely to experience SJS/TEN during anticancer therapy, with ORs of 1.419 (95% CI: 1.115–1.805, *p* = 0.003) and 0.842 (95% CI: 0.748–0.946, *p* = 0.004), respectively. Additionally, regarding anticancer drug types, the following 10 therapeutics—pembrolizumab, nivolumab, carboplatin, enfortumab vedotin, ipilimumab, bendamustine, atezolizumab, vemurafenib, pemetrexed, and apalutamide—were identified as independent risk factors for anticancer drug-associated SJS/TEN (*p* < 0.01). Model performance was evaluated through receiver operating characteristic (ROC) curve analysis, yielding an area under the curve (AUC) of 0.70 (**Figure 4C**), which suggests the predictive accuracy of the anticancer-associated SJS/TEN risk assessment model is clinically acceptable.

**Table 2:** Multivariable logistic regression analysis.

Variables	Coef.	SE	z.value	p.value	OR (95%CI)
Intercept	-5.926	0.096	-61.576	< 0.001*	0.003 (0.002-0.003)
Age					
≥ 65 years	0.35	0.12	2.917	0.003*	1.419 (1.115-1.805)
Gender					
Male	-0.172	0.06	-2.874	0.004*	0.842 (0.748-0.946)
Drugs					
Pembrolizumab	1.83	0.109	16.847	< 0.001*	6.231 (5.008-7.669)
Nivolumab	1.248	0.116	10.785	< 0.001*	3.483 (2.757-4.342)
Carboplatin	0.814	0.143	5.71	< 0.001*	2.256 (1.687-2.952)
Enfortumab Vedotin	3.985	0.184	21.614	< 0.001*	53.76 (36.815-76.003)
Ipilimumab	1.344	0.204	6.584	< 0.001*	3.833 (2.502-5.589)
Bendamustine	1.84	0.201	9.167	< 0.001*	6.299 (4.144-9.134)
Atezolizumab	0.919	0.187	4.927	< 0.001*	2.507 (1.702-3.546)
Vemurafenib	2.264	0.198	11.461	< 0.001*	9.618 (6.375-13.869)
Pemetrexed	1.665	0.187	8.881	< 0.001*	5.285 (3.582-7.489)
Apalutamide	2.589	0.238	10.885	< 0.001*	13.311 (8.072-20.61)

OR, odds ratio; CI, confidence interval. The analysis was adjusted for age (reference: <65 years), gender (reference: female), and drugs (reference: all other anticancer drugs). * *p* < 0.01.

### 3.5 Time onset analysis of anticancer therapy-related SJS/TEN

A total of 1662 reports indicated the specific onset time of SJS/TEN development associated with anticancer therapy in the FAERS database (**Table 3**). As illustrated in **Supplementary Figure 2**, the majority of SJS/TEN associated with anticancer drugs developed within the initial month of treatment (n=1076, 64.74%). The median onset time of anticancer agents induced SJS/TEN was 17 days (IQR: 7 – 48) (**Table 3**). Furthermore, anticancer drugs with a statistically significant association with SJS/TEN development and with more than 20 reported cases were subjected to further analysis of time to onset. The time to onset of SJS/TEN associated with different anticancer therapies differed significantly. Data in **Table 3** demonstrate that the median onset time was the shortest at 9.5 days (IQR: 6.25 – 12) for sorafenib and the longest at 35 days (IQR: 15 – 55) for bendamustine. As shown by the results of the Weibull shape parameter analysis, for most of the statistically significant anticancer drugs, the 95% upper limit of the shape parameter β is less than 1, which belongs to the early failure type, indicating that the risk initially increases and then decreases. Enfortumab vedotin, bendamustine, and pemetrexed, however, were randomized failures (95% CI for β included 1), indicating consistent risks throughout the exposure period. In addition, for vemurafenib and apalutamide, the risks of SJS/TEN increased over time (wear-out failure type, with β > 1).

**Table 3:** Time-to-onset analysis of SJS/TEN associated with anticancer therapies in the FAERS database.

Drugs	Cases^*^	TTO (days)		Webibull distribution				Failure type
				Scale parameter		Shape parameter		
	n	Median	IQR	α	95%CI	β	95%CI	
Anti-cancer drugs	1662	17	7-48	42.00	38.56-45.43	0.64	0.62-0.66	Early Failure
Pembrolizumab	135	32	13-82.5	65.29	50.28-80.30	0.79	0.69-0.89	Early Failure
Nivolumab	124	27	13-60.75	58.48	43.29-73.67	0.74	0.65-0.83	Early Failure
Carboplatin	97	31	10-64	49.73	37.43-62.02	0.86	0.73-0.98	Early Failure
Enfortumab Vedotin	77	14	10-21	24.97	18.35-31.59	0.91	0.77-1.05	Random Failure
Ipilimumab	41	19	9-41	35.94	19.30-52.57	0.70	0.55-0.85	Early Failure
Bendamustine	31	35	15-55	55.80	35.54-76.07	1.06	0.79-1.34	Random Failure
Atezolizumab	47	18	9.5-85	54.26	30.18-78.34	0.68	0.54-0.83	Early Failure
Vemurafenib	36	14	8.75-23.25	17.57	14.13-21.02	1.75	1.30-2.21	Wear-out Failure
Pemetrexed	50	25	6-92	54.91	34.27-75.56	0.83	0.65-1.02	Random Failure
Apalutamide	26	30	21-40.5	37.32	28.81-45.83	1.85	1.30-2.40	Wear-out Failure
Gemcitabine	32	12.5	3.75-36.25	34.92	16.82-53.01	0.73	0.54-0.93	Early Failure
Temozolomide	32	18.5	6.75-53.25	38.79	20.04-57.54	0.76	0.56-0.96	Early Failure
Sorafenib	46	9.5	6.25-12	17.51	10.07-24.96	0.73	0.60-0.87	Early Failure

* Only cases that reported a specific time of SJS/TEN onset and had a sample size greater than 20 were included in the time-to-onset analysis.α, scale parameter, represents the scale of the distribution function as the quantile in which 63.2% of AEs occur. β, shape parameter, could be used to confirm the distribution type: early failure type (β < 1), random failure type (95% CI of β include 1), and wear-out type (β > 1).

## 4 Discussion

Over the past few decades, the traditional paradigm of cancer treatment has been revolutionized with the approval of novel anticancer agents, which not only benefit patient survival but also improve their quality of life (21). Conventional chemotherapy, along with targeted or immunotherapies, is considered well tolerated, but may give rise to a variety of cutaneous AEs, spanning from non-life-threatening cutaneous toxicities such as paronychia, alopecia, and acneiform eruption to life-threatening severe cutaneous AEs such as SJS and TEN (12, 22). Given the low incidence of SJS/TEN in patients receiving anticancer therapies, early randomized controlled trials may not be able to fully monitor the corresponding safety risks (13, 23). This pharmacovigilance study systematically quantifies the risks of SJS/TEN across classes of anticancer agents by using a large number of post-marketing samples in the FAERS database. Utilizing the analysis of risk factors, we aim to demarcate patient subgroups with elevated susceptibility profiles, ultimately generating evidence-based risk metrics to optimize therapeutic decision-making in oncology practice.

This study identified 3471 distinct significant associations between SJS/TEN events and anticancer drugs, involving 159 unique anticancer agents (**Supplementary Table S2**). As indicated in **Table 1**, the median age of patients who developed SJS/TEN associated with anticancer drug use was 64 years (IQR: 52–71 years), indicating a higher prevalence of SJS/TEN among middle-aged and elderly populations. In a population-based longitudinal cohort study encompassing 2,398,393 Japanese individuals, advanced age was identified as a significant risk factor for the development of SJS/TEN (24). The findings of our study are in congruence with prior research, which suggests that elderly individuals are more susceptible to polypharmacy and experience an age-associated decline in immune function (25–27). As presented in **Figure 1A**, between 2004 and the third quarter of 2024, there was an almost tenfold increase in the reported incidences of SJS/TEN associated with anticancer drugs, reflecting a steady upward tendency. While the heightened awareness of AE reporting likely contributed to this rise, it is also plausible that the clinical application of novel anticancer agents has played a significant role. Moreover, it is not difficult to see from our research that reports of anticancer drug-related SJS/TEN are more frequently from the United States, Japan, and France (**Figure 1B**), which may reflect the active pharmacovigilance systems in these countries or differences in susceptibility to SJS/TEN in the population due to genetic polymorphisms in human leukocyte antigen (HLA) (28, 29).

Hitherto, SJS/TEN represent a significant clinical and economic burden worldwide. Notably, the mortality rate in patients with these events can be as high as 50% (30). In our large-scale cohort study, although the mortality and hospitalization rates associated with anticancer drug-induced SJS/TEN were lower than previously reported, they remained alarmingly high. Specifically, the mortality rate was 32.0% and the hospitalization rate was 30.9%, underscoring the serious clinical implications of these adverse drug reactions. Drugs, considered as foreign antigens, likely interact with particular HLA/peptide/T-cell receptor (TCR) complexes on keratinocytes to initiate an adaptive immune response, ultimately leading to cutaneous AEs, including SJS/TEN (30, 31). Given this immunopathogenic mechanism, prompt identification of the causative drug and its immediate discontinuation represent critical initial steps in the management of patients with SJS/TEN.

Our disproportionality study revealed potential associations of anticancer drugs with SJS/TEN, including conventional cytotoxic and novel targeted agents. As depicted in **Figure 2**, the distribution of SJS/TEN cases within the FAERS dataset varied slightly across four distinct anticancer drug classes. Among the 159 anticancer drugs linked to the development of SJS/TEN, targeted therapy, chemotherapy, immunotherapy, and endocrine therapy accounted for 35.93%, 35.52%, 21.52%, and 5.50% of the associated cases, respectively.

Chemotherapy serves as the cornerstone of anticancer drug regimens and is also the most widely used type of medication in the field of oncology. A previous systematic review revealed that docetaxel and methotrexate were the most common drugs to cause chemotherapy-related SJS/TEN (32). In the present study, the most common chemotherapies that induced SJS/TEN were methotrexate (n = 232) and carboplatin (n = 153) (**Supplementary Table S2**), and the most significant disproportionate chemotherapies that induced SJS/TEN were carboplatin (n = 153) and bendamustine (n = 86) (**Figure 3**). For carboplatin, platinum-DNA complexes induce an immune response that damages keratinocytes, which in turn disrupts the skin-mucosal barrier, resulting in the initiation of SJS/TEN (11). Although methotrexate showed no significant signal for SJS/TEN in our analysis, this does not mean that it is risk-free. It merely suggests that such AEs do not appear to be disproportionately associated with methotrexate (33). Moreover, both antimetabolites and alkylating agents (e.g., methotrexate, cytarabine, 5-fluorouracil, mercaptopurine) can induce toxic erythema. This condition is characterized by minimal inflammatory infiltrates, closely mimicking SJS/TEN, but exhibits distinct clinicopathological features (32, 34). By contrast, SJS/TEN is an allergic reaction driven by cytotoxic T lymphocytes and natural killer cells. Therefore, accurate clinical differentiation and pathological confirmation between chemotherapy-associated SJS/TEN and toxic erythema are crucial, as inappropriate management can significantly impact patient outcomes.

Targeted anticancer therapies and immunotherapies - potent new classes of pharmaceutical agents - have emerged as pivotal treatment modalities for diverse cancer types. Evidence from previous systematic reviews suggested that imatinib, vemurafenib, and rituximab are the top three targeted therapies that most commonly cause SJS/TEN, while epidermal growth factor receptor inhibitors are the class of agents that most commonly induce SJS/TEN (35). Consistent with previous findings, our pharmacovigilance analysis revealed that, in terms of quantity, rituximab (n = 110, ROR025 = 0.58) and vemurafenib (n = 72, ROR025 = 3.75) remain the primary targeted therapies for the triggering of SJS/TEN (**Supplementary Table S2**; **Figure 3**). BRAF inhibitor treatment has emerged as a standard of management for patients with advanced BRAF V600-mutant melanoma, but the mechanism of SJS/TEN associated with BRAF inhibitor therapy remains incompletely understood. A previous study reported that patients who began receiving vemurafenib after ipilimumab use may be at a greater risk of developing SJS/TEN (36). This might be explained by the strong provocation of CD8^+^ cytotoxic T cell activation by ICIs, as these cells also serve as key cellular mediators in the pathogenesis of SJS/TEN (37). ICIs have been the focus of much attention in recent years, and indeed, many cases of SJS/TEN in patients treated with ICIs have been previously reported (38–40). In our disproportionality analysis, immunotherapy-related SJS/TEN accounted for 21.52% of all anticancer treatments (n=747) (**Figure 2**). One proposed mechanism for ICIs-associated SJS/TEN involves ICIs-induced cytotoxicity, whereby activated T cells target keratinocytes, ultimately prompting their apoptotic death (35, 41, 42). Currently, the consensus recommendation is to permanently discontinue the offending drug along with the application of systemic immunomodulatory drugs that may be of benefit to the patient, such as pulse steroids. Therefore, oncologists, dermatologists, and pharmacists are encouraged to collaborate closely to optimize therapeutic strategies, as immunomodulatory medications may interfere with the efficacy of ICIs (43).

The present study, like other pharmacovigilance studies based on spontaneous reporting systems, acknowledged certain limitations. First, FAERS is a passive reporting system and as such is subject to many biases such as under-reporting, over-reporting, or selectivity in reporting. Second, these findings are derived from retrospective data, which inherently carry the potential for confounding variables. For example, patients undergoing anticancer therapy are often prescribed adjuvant medications (e.g., analgesics, antiemetics, antibiotics) in conjunction with their oncology regimens, several of which—including allopurinol, sulfonamide antibiotics, anticonvulsants, and corticosteroids—have well-documented risks of inducing SJS/TEN. Third, the FAERS reports did not provide sufficiently detailed information (e.g., disease severity, clinical, laboratory, or histological data of SJS/TEN) to facilitate an accurate diagnosis of SJS/TEN. Finally, the disproportionality signals do not imply causality but warrant further investigation due to elevated reporting rates. Moreover, although we considered numerous variables influencing SJS/TEN, the absence of key data—such as specific drug dosages and treatment durations—limited the depth of our analysis. The explanatory power of our multivariate logistic regression model, as indicated by MacFadden’s R² value of 0.115, accounted for only a small portion of the variability in the results. This limitation highlights the need for future studies to increase the sample size and incorporate additional predictive factors to enhance model accuracy and better elucidate the complexity of anticancer therapies related SJS/TEN events. Taking these limitations into account, the results of this study should be interpreted carefully.

Despite these limitations, the present study has three key strengths: first, it utilizes the FAERS database, an extensive spontaneous reporting system that has collected global, non-controlled, real-world clinical reports over a 20-year period; second, its methodology not only confirms established associations but also detects emerging SJS/TEN safety signals for both long-prescribed anticancer drugs and newly approved agents, including the identification of 7 high-signal drugs lacking current label warnings; and third, its scope encompasses SJS/TEN risk profiles across all major anticancer drug classes, with stratification of time-to-onset patterns supported by Weibull β estimates. This study establishes a framework for the timely identification of treatment-related SJS/TEN toxicity risks associated with specific anticancer drugs. Based on the data obtained for these anticancer agents, we believe these findings will enhance understanding of SJS/TEN toxicity risks linked to anticancer therapies, thereby enabling clinicians to implement preventive mitigation strategies or consider alternative treatment regimens when clinically indicated.

## 5 Conclusion

This study was the first to systematically uncover the potential relationship between the burden of and all major classes of anticancer therapeutics using the FAERS database. Insights from the FAERS database revealed that 31 anticancer drugs were identified as significantly disproportionate to the development of SJS/TEN, whereas the package inserts for 7 of these drugs do not include warnings about the risk of SJS/TEN. The median onset of SJS/TEN with anticancer therapies was 17 days. Moreover, the results of multivariable logistic regression analysis showed that age above 65 years and male were significant risk factors for the incidence of anticancer therapies-related SJS/TEN. Given the expanding indications of novel anticancer agents, further research into the mechanisms and optimal management strategies for anticancer therapies-related SJS/TEN is needed. Addressing this knowledge gap will facilitate the development of improved prevention and treatment strategies, optimize clinical management, and support evidence-based, patient-centered decision making.

## Data Availability

The original contributions presented in the study are included in the article/**Supplementary Material**. Further inquiries can be directed to the corresponding authors.
