# Supplementary figures and images for: Emerging causes of anticancer therapies−induced Stevens-Johnson syndrome and toxic epidermal necrolysis: evidence from disproportionality analysis of the FDA adverse event reporting system
Source: Front Immunol. 2025 Aug 27;16:1646038. doi: 10.3389/fimmu.2025.1646038 (PMC12420621; doi:10.3389/fimmu.2025.1646038)

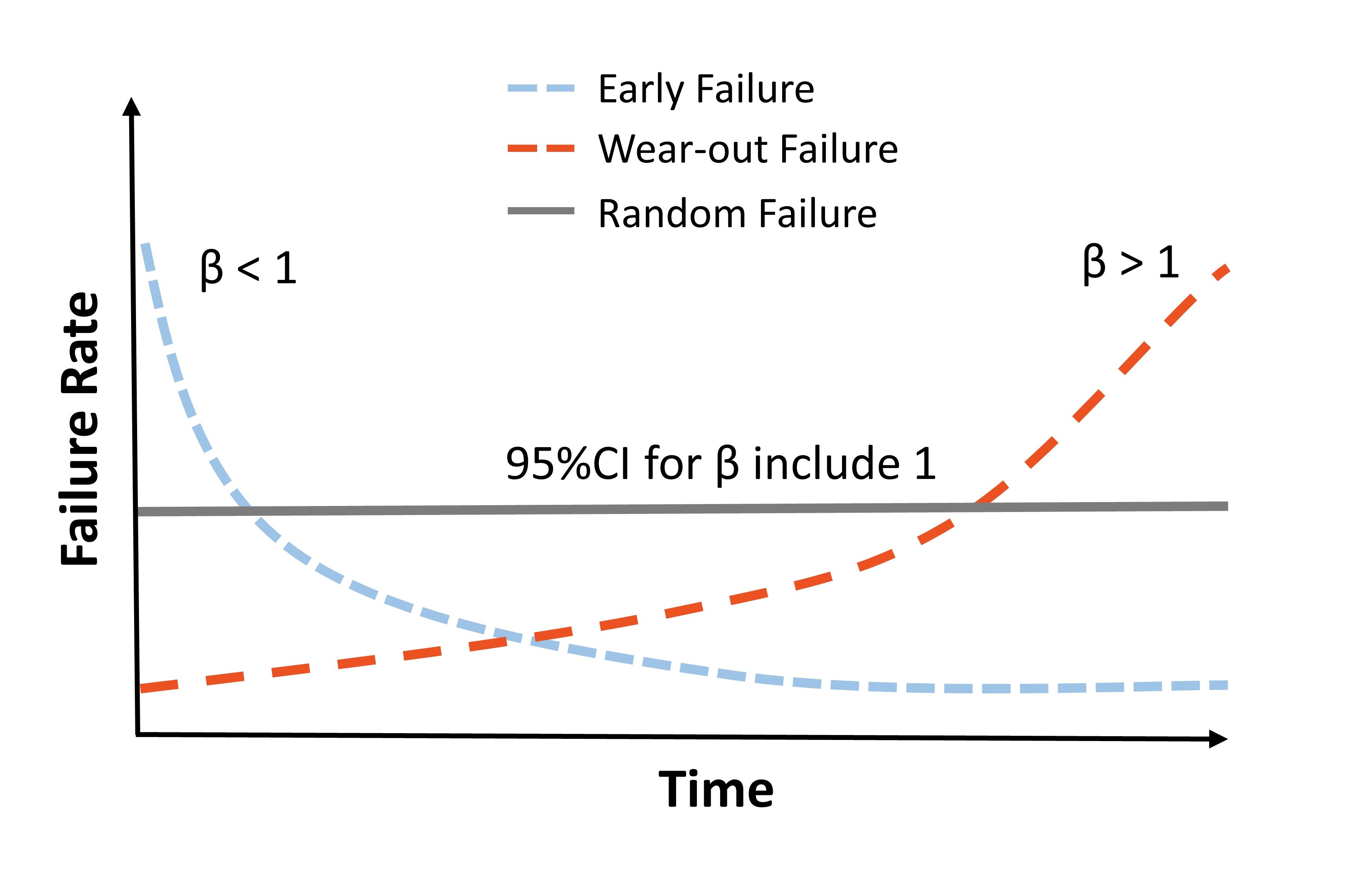

Supplement: Supplementary file 1 [file Image1.jpeg]
