# Supplementary material for: Emerging causes of anticancer therapies−induced Stevens-Johnson syndrome and toxic epidermal necrolysis: evidence from disproportionality analysis of the FDA adverse event reporting system
Source: Front Immunol. 2025 Aug 27;16:1646038. doi: 10.3389/fimmu.2025.1646038 (PMC12420621; doi:10.3389/fimmu.2025.1646038)
Supplement: Supplementary file 3 [file Table1.docx]

**Supplementary Table 1** Two - by - two contingency table for disproportionality analysis.

|  | **Number of SJS/TEN events** | **Number of other adverse events** | **Total** |
| --- | --- | --- | --- |
| **Target drugs** | a | b | a + b |
| **Other drugs** | c | d | c + d |
| **-Total** | a + c | b + d | a + b + c + d |

Note: a, number of SJS/TEN events of Target drugs alone; b, number of other AEs of Target drugs alone; c, number of SJS/TEN events of other drugs except for anticancer therapies; d, number of other AEs of other drugs except for anticancer therapies.
